# Incidence and Progression of Alcohol-Associated Liver Disease After Medical Therapy for Alcohol Use Disorder

**DOI:** 10.1001/jamanetworkopen.2022.13014

**Published:** 2022-05-20

**Authors:** Augustin G. L. Vannier, Jessica E. S. Shay, Vladislav Fomin, Suraj J. Patel, Esperance Schaefer, Russell P. Goodman, Jay Luther

**Affiliations:** 1 Alcohol Liver Center, Massachusetts General Hospital, Harvard Medical School, Boston; 2 Gastrointestinal Unit, Department of Medicine, Massachusetts General Hospital, Harvard Medical School, Boston; 3 Department of Medicine, Massachusetts General Hospital, Harvard Medical School, Boston; 4 Department of Medicine, University of Texas at Southwestern, Dallas

## Abstract

**Importance:**

Alcohol-associated liver disease (ALD) is one of the most devastating complications of alcohol use disorder (AUD), an increasingly prevalent condition. Medical addiction therapy for AUD may play a role in protecting against the development and progression of ALD.

**Objective:**

To ascertain whether medical addiction therapy was associated with an altered risk of developing ALD in patients with AUD.

**Design, Setting, and Participants:**

This retrospective cohort study used the Mass General Brigham Biobank, an ongoing research initiative that had recruited 127 480 patients between its start in 2010 and August 17, 2021, when data for the present study were retrieved. The mean follow-up duration from AUD diagnosis was 9.2 years. *International Statistical Classification of Diseases and Related Health Problems, Tenth Revision* diagnosis codes were used to identify ALD and AUD diagnoses.

**Exposures:**

Medical addiction therapy was defined as the documented use of disulfiram, acamprosate, naltrexone, gabapentin, topiramate, or baclofen. Patients were considered to be treated if they initiated medical addiction therapy before the relevant outcome.

**Main Outcomes and Measures:**

Adjusted odds ratios (aORs) for the development of ALD and hepatic decompensation were calculated and adjusted for multiple risk factors.

**Results:**

The cohort comprised 9635 patients with AUD, of whom 5821 were male individuals (60.4%), and the mean (SD) age was 54.8 (16.5) years. A total of 1135 patients (11.8%) had ALD and 3906 patients (40.5%) were treated with medical addiction therapy. In multivariable analyses, medical addiction therapy for AUD was associated with decreased incidence of ALD (aOR, 0.37; 95% CI, 0.31-0.43; *P* < .001). This association was evident for naltrexone (aOR, 0.67; 95% CI, 0.46-0.95; *P* = .03), gabapentin (aOR, 0.36; 95% CI, 0.30-0.43; *P* < .001), topiramate (aOR, 0.47; 95% CI, 0.32-0.66; *P* < .001), and baclofen (aOR, 0.57; 95% CI, 0.36-0.88; *P* = .01). In addition, pharmacotherapy for AUD was associated with lower incidence of hepatic decompensation in patients with cirrhosis (aOR, 0.35; 95% CI, 0.23-0.53, *P* < .001), including naltrexone (aOR, 0.27; 95% CI, 0.10-0.64; *P* = .005) and gabapentin (aOR, 0.36; 95% CI, 0.23-0.56; *P* < .001). This association persisted even when medical addiction therapy was initiated only after the diagnosis of cirrhosis (aOR, 0.41; 95% CI, 0.23-0.71; *P* = .002).

**Conclusions and Relevance:**

Results of this study showed that receipt of medical addiction therapy for AUD was associated with reduced incidence and progression of ALD. The associations of individual pharmacotherapy with the outcomes of ALD and hepatic decompensation varied widely.

## Key Points

**Question:**

Are patients with alcohol use disorder (AUD) who receive medical addiction therapy less likely to develop alcohol-associated liver disease (ALD)?

**Findings:**

In this cohort study of 9635 patients with AUD, those who received medical addiction therapy had a significantly lower risk of developing ALD, whereas patients with cirrhosis who received medical addiction therapy had a significantly lower incidence of hepatic decompensation.

**Meaning:**

Findings from this study suggest an association between use of medical addiction therapy for AUD and decreased incidence and progression of ALD.

## Teaser

This cohort study assesses the association between pharmacotherapy and the development of alcohol-associated liver disease in patients with alcohol use disorder.

## Introduction

Alcohol-associated liver disease (ALD) is among the most common and devastating complications of excessive alcohol use.^1^ Alcohol-associated liver disease represents a wide spectrum, ranging from the relatively benign hepatic steatosis to cirrhosis and hepatocellular carcinoma.^2^ The most aggressive form of ALD is severe alcohol-associated hepatitis, which has a 30% 3-month mortality rate.^3,4^ A surge in the incidence of ALD is expected with the COVID-19–related increase in alcohol use.^5,6,7^ It is, therefore, critical to prevent the development of ALD to limit the morbidity and mortality associated with excessive alcohol use.

Some medical treatments for alcohol use disorder (AUD) exist. Disulfiram, acamprosate, and naltrexone are 3 US Food and Drug Administration (FDA)–approved medical addiction therapy medications for AUD that have been associated with improved rates of abstinence, reduced binge drinking, and decreased rate of AUD-related hospitalizations.^8,9,10,11^ Furthermore, in patients with AUD, gabapentin and topiramate have been associated with a decreased number of days of heavy drinking,^12,13^ whereas baclofen has shown promise as a factor in achieving abstinence.^14^

Data are limited on the use of medical addiction therapy for AUD in patients with ALD. Baclofen has been the most well examined in this context, with multiple studies showing its favorable safety profile and potential benefit in patients with ALD.^15,16,17,18,19^ More recent studies found that patients with cirrhosis who received FDA-approved therapies for AUD were less likely to experience hepatic decompensation over a 6- to 12-month period.^20,21^

Despite these intriguing findings, certain questions remain. First, does medical addiction therapy play a role in preventing the development of ALD in patients with AUD? Second, do off-label medications for medical addiction therapy (gabapentin, topiramate, and baclofen) provide a benefit for patients with ALD-associated cirrhosis? Third, is there a long-term benefit (>1 year) with regard to protection against hepatic decompensation in patients with cirrhosis receiving medical addiction therapy?

In this study, we leveraged a well-characterized cohort of patients with AUD who had long-term follow-up data to ascertain whether medical addiction therapy was associated with an altered risk of developing ALD. In addition, we sought to ascertain whether medical addiction therapy was associated with reduced risk of hepatic decompensation in patients with alcohol-associated cirrhosis. We analyzed both FDA-approved and off-label pharmacotherapy for AUD.

## Methods

For this retrospective cohort study, we identified eligible patients participating in the Mass General Brigham Biobank,^22^ an ongoing research initiative that had recruited 127 480 patients between its start in 2010 and August 17, 2021, when we retrieved data for this study. All patients included in the Biobank database provided written informed consent. Because all data from the database are deidentified and contain no protected health information, the study protocol was deemed exempt by the institutional review board of Massachusetts General Hospital. We followed the Strengthening the Reporting of Observational Studies in Epidemiology (STROBE) reporting guideline.

We retrieved retrospective data, with the earliest AUD diagnosis occurring in 1979 and the most recent in 2021. The mean follow-up duration from AUD diagnosis was 9.2 years. Demographic, clinical, and social data were available for each patient and accessible through the online platform of the Biobank. Data in the Biobank were derived from the electronic health record of Mass General Brigham. To identify patients with AUD, we queried the Biobank database for patients with the following *International Statistical Classification of Diseases and Related Health Problems, Tenth Revision (ICD-10)* diagnosis codes: alcohol abuse (F10.1) or alcohol dependence (F10.2).

### Demographic and Clinical Variables

We collected the following demographic and clinical data: age, sex, race and ethnicity, history of homelessness, body mass index (BMI; calculated as weight in kilograms divided by height in meters squared), viral hepatitis status, history of nonalcohol substance use disorder and nicotine dependence, history of psychiatric disorder and receipt of nonpharmaceutical psychotherapy, and other liver diseases. Race and ethnicity were assessed because they may modulate the likelihood of developing ALD. Race and ethnicity data were derived from the electronic health record of Mass General Brigham and were categorized as follows: Asian, Black, Hispanic, White, other, or unknown.

Viral hepatitis was assessed using laboratory testing results and *ICD-10* diagnosis codes. Patients were classified as having hepatitis C if they had a positive hepatitis C antibody test result and as having hepatitis B if they had a positive hepatitis B antigen test result or had a diagnosis of either infection. Patients who were missing either a hepatitis C antibody test result or a hepatitis B antigen test result and did not have a diagnosis of viral hepatitis were considered to be untested for the relevant infection in the multivariable analyses. A BMI lower than 25 was categorized as nonoverweight, between 25 and 29.9 as overweight, between 30 and 34.9 as class I obesity, between 35 and 39.9 as class II obesity, and 40 or higher as class III obesity. Patients with missing BMI information were labeled as having unknown BMI in the multivariable analyses.

We also collected data on other risk factors for AUD (eTable 1 in the Supplement). A list of the *ICD-10* diagnosis codes, *Current Procedural Terminology* codes, and test results that were used to define all variables in the multivariable analyses is provided in eTable 1 in the Supplement.

### Definitions of ALD and Addiction Treatment 

We identified patients with ALD who had at least 1 of the following *ICD-10* diagnosis codes: alcoholic hepatitis (K70.1), alcoholic cirrhosis of liver (K70.3), alcoholic hepatic failure (K70.4), alcoholic fibrosis and sclerosis (K70.2), other cirrhosis (K74.69), or unspecified cirrhosis (K74.60). We did not consider patients to have liver disease if they had a diagnosis of either alcoholic fatty liver (K70.0) or alcoholic liver disease–unspecified (K70.9) because many patients with moderate and high alcohol use develop hepatic steatosis, which has unclear clinical significance regarding liver-related morbidity and mortality.^23^

We studied several medications for AUD: disulfiram, acamprosate, naltrexone, gabapentin, baclofen, and topiramate. To narrow the focus on patients who received sustained pharmacotherapy, we included patients who received at least 3 prescriptions of a given pharmacotherapy. Patients were considered to be treated in each analysis if they initiated medical addiction therapy before the relevant outcome. Patients were considered to be untreated if they received no medical addiction therapy before the relevant outcome. The duration of a given therapy was the time between the first and the most recent prescription of medical addiction therapy. We also collected data on psychotherapy for AUD and accounted for them in the multivariable analyses (eTable 1 in the Supplement).^24^

### Statistical Analysis

Continuous variables were summarized with means (SDs) and compared using an unpaired, 2-tailed *t* test with Welch correction, whereas categorical variables were compared using the Fisher exact test. We also performed multivariable logistic regressions to calculate the adjusted odds ratios (aORs) with 95% CIs of developing ALD or hepatic decompensation, adjusting for multiple factors, such as demographic variables (age, sex, and race and ethnicity), history of homelessness, individual psychiatric disorders, nonalcohol substance use disorders, receipt of nonpharmaceutical psychotherapy, and concurrent liver diseases (positive test result for hepatitis B or C virus, nonalcoholic steatohepatitis, obesity, primary sclerosing cholangitis, primary biliary cholangitis, autoimmune hepatitis, chronic passive liver congestion, α_1_-antitrypsin deficiency, or hemochromatosis) (eTable 1 in the Supplement). When we examined individual medical addiction therapy, we accounted for the receipt of other such treatments by including in the statistical model every pharmacotherapy for addiction received by a patient; thus, the associations for an individual drug were independent of other medical addiction therapy prescriptions.

In addition, we used Cox proportional hazards regression model to account for the follow-up duration, generating hazard ratios (HRs) for the development and progression of ALD. To minimize immortal time bias in patients who were treated, we counted follow-up from either medical addiction therapy initiation or meeting of an inclusion criterion (AUD diagnosis or cirrhosis diagnosis), whichever occurred more recently. The Cox proportional hazards regression models and logistic regressions were adjusted for all variables in eTable 1 in the Supplement. We also used a Kaplan-Meier analysis to examine the proportion of patients over time who developed hepatic decompensation after an index cirrhosis diagnosis.

All analyses were conducted using GraphPad Prism (GraphPad Software). *P* < .05 was considered to be statistically significant in all analyses. All statistical tests were 2-tailed.

## Results

**Figure 1.**
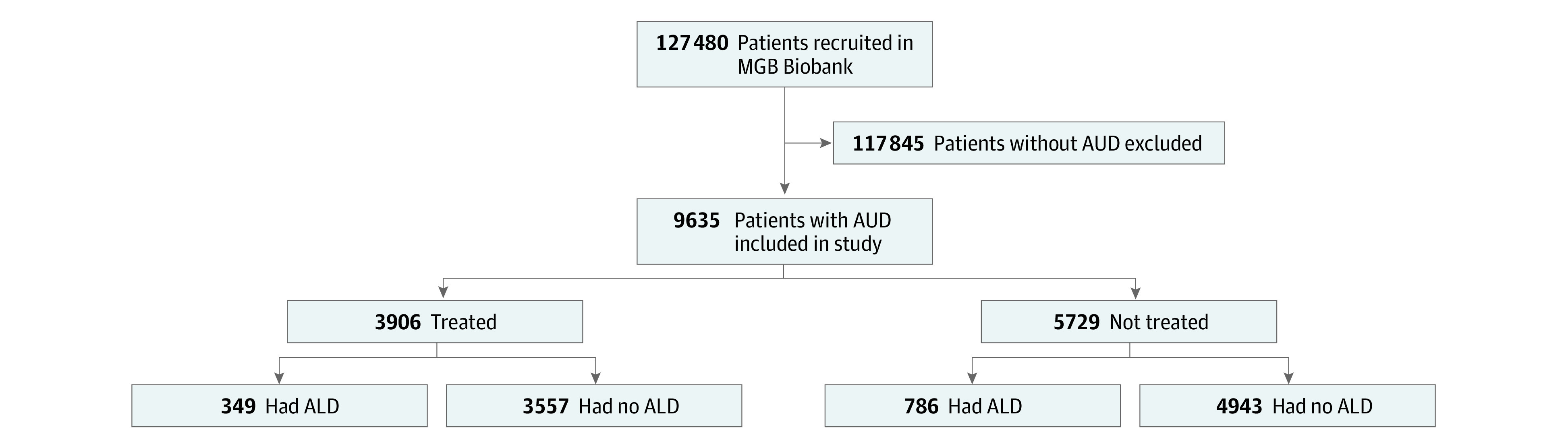
Flowchart of Patient Selection Patients with alcohol use disorder (AUD) were considered to be treated if they received 3 prescriptions for at least 1 of the following: disulfiram, acamprosate, naltrexone, gabapentin, topiramate, or baclofen. ALD indicates alcohol-associated liver disease; MGB, Mass General Brigham.

We identified 9635 patients with AUD, of whom 5821 were male (60.4%) and 3814 were female (39.5%) individuals with a mean (SD) age of 54.8 (16.5) years; most patients (8045 [83.4%]) had White race (Table 1). Of the patients with AUD, 1135 had ALD (11.8%) and 3906 (40.5%) were treated with medical addiction therapy (Figure 1). Patients with AUD in the treated group had different demographic characteristics compared with patients in the untreated group (Table 1; eTable 2 in the Supplement).

**Table 1.** Demographic and Social Characteristics of Patients by Group

Characteristic	No. (%)			*P* value^a^
	All patients	Medical addiction therapy		
		Treated group	Untreated group	
No.	9635 (100)	3906 (40.5)	5729 (59.5)	NA
Age, mean (SD), y	54.8 (16.5)	55.0 (15.3)	54.7 (17.3)	.26
Sex				
Female	3814 (39.5)	1736 (44.4)	2078 (36.2)	<.001
Male	5821 (60.4)	2170 (55.5)	3651 (63.7)	<.001
Race and ethnicity^b^				
Asian	19 (0.3)	19 (0.4)	65 (1.1)	<.001
Black	288 (5.0)	288 (7.3)	432 (7.5)	.78
Hispanic	108 (1.8)	108 (2.7)	212 (3.7)	.01
White	8045 (83.4)	3313 (84.8)	4732 (82.5)	<.001
Other^c^	132 (2.3)	132 (3.3)	200 (3.4)	.78
Unknown	154 (2.6)	154 (3.9)	300 (5.2)	.003
BMI, mean (SD)	28.7 (6.7)	29.2 (6.4)	28.4 (7.0)	<.001
Viral hepatitis^d^	1204 (12.4)	635 (16.2)	569 (9.9)	<.001
History of homelessness	978 (10.1)	604 (15.4)	374 (6.5)	<.001
Receipt of psychotherapy	3722 (38.6)	1977 (50.6)	1745 (30.4)	<.001
History of psychiatric disorder	8227 (85.3)	3674 (94.0)	4553 (79.4)	<.001
History of nicotine dependence	4260 (44.2)	2113 (54.0)	2147 (37.4)	<.001
Nonalcohol SUD^e^	3532 (36.6)	1947 (49.8)	1585 (27.6)	<.001

Abbreviations: BMI, body mass index (calculated as weight in kilograms divided by height in meters squared); NA, not applicable; SUD, substance use disorder.

^a^ *P* values for categorical variables were the results of a Fisher exact test comparing patients receiving any medical addiction therapy with those receiving none. *P* values for continuous variables were the results of an unpaired, 2-tailed *t* test with Welch correction.

^b^ Race and ethnicity data were derived from the electronic health record of Mass General Brigham.

^c^ Information on other category was not available.

^d^ Positive test result or diagnosis.

^e^ Nonalcohol SUD involved cannabis, cocaine, other stimulant, opioid, inhalant, or sedative.

### Incidence of ALD After Medical Addiction Therapy

We sought to ascertain whether the initiation of medical addiction therapy for AUD was associated with reduced risk of developing ALD in the future. The mean duration of therapy for patients in the treated group was 4.1 years, whereas the mean follow-up duration from AUD diagnosis was 9.8 years in the treated group and 8.8 years in the untreated group (eFigure in the Supplement). Patients in the treated group initiated treatment a mean (SD) 1.65 (6.4) years after their index AUD diagnosis. For patients who were treated and developed ALD, the first ALD diagnosis occurred a mean (SD) 4.8 (4.3) years after medical addiction therapy initiation.

In a multivariable analysis, we found that the receipt of any pharmacotherapy for AUD was independently associated with decreased incidence of ALD (aOR, 0.37; 95% CI, 0.31-0.43; *P* < .001) (Table 2). When accounting for the time of follow-up using Cox proportional hazards regression, we found that the receipt of any pharmacotherapy for AUD was associated with reduced odds of developing ALD (HR, 0.76; 95% CI, 0.64-0.0.89; *P* < .001). Furthermore, we found a dose-dependent association: among patients in the treated group, each year of treatment was associated with reduced likelihood of developing ALD (HR, 0.97; 95% CI, 0.95-0.99; *P* = .04).

**Table 2.** Odds Ratios for the Development of Alcohol-Associated Liver Disease After Medical Addiction Therapy

Medical addiction therapy	Adjusted odds ratio (95% CI)	*P* value
Any pharmacotherapy	0.37 (0.31-0.43)	<.001
Gabapentin	0.36 (0.30-0.43)	<.001
Topiramate	0.47 (0.32-0.66)	<.001
Baclofen	0.57 (0.36-0.88)	.01
Naltrexone	0.67 (0.46-0.95)	.03
Disulfiram	0.86 (0.43-1.61)	.66
Acamprosate	2.59 (1.84-3.61)	<.001

Receipt of gabapentin (aOR, 0.36; 95% CI, 0.30-0.43; *P* < .001), topiramate (aOR, 0.47; 95% CI, 0.32-0.66; *P* < .001), baclofen (aOR, 0.57; 95% CI, 0.36-0.88; *P* = .01), and naltrexone (aOR, 0.67; 95% CI, 0.46-0.95; *P* = .03) was independently associated with decreased odds of developing ALD (Table 2). Treatment with disulfiram was not associated with an outcome, whereas acamprosate was associated with increased odds of ALD diagnosis (aOR, 2.59; 95% CI, 1.84-3.61; *P* < .001).

### Incidence of Hepatic Decompensation After Medical Addiction Therapy

Having found that medical addiction therapy for AUD was independently associated with decreased incidence of ALD, we investigated the association between medical addiction therapy and the progression of ALD. After the index cirrhosis diagnosis, patients in the treated group were followed up for a mean (SD) duration of 7.8 (6.5) years and patients in the untreated group were followed up for a mean (SD) duration of 8.6 (6.3) years and then examined for incident hepatic decompensation. Patients in the treated group initiated treatment a mean (SD) 1.46 (5.9) years before their index cirrhosis diagnosis. Among patients who were treated and experienced a hepatic decompensating event, the first decompensation occurred a mean (SD) 5.1 (4.7) years after the initiation of medical addiction therapy. In a multivariable analysis, we found that the receipt of any pharmacotherapy for AUD was associated with reduced incidence of a hepatic decompensating event (aOR, 0.35; 95% CI, 0.23-0.53; *P* < .001) (Table 3). In a Cox proportional hazards regression that accounted for the duration of follow-up, we found that patients in the treated group were less likely to experience a hepatic decompensating event compared with patients in the untreated group (HR, 0.45; 95% CI, 0.34-0.60; *P* < .001). Each year of medical addiction therapy was associated with further decrease in the likelihood of hepatic decompensation (HR, 0.93; 95% CI, 0.88-0.99; *P* = .02).

**Table 3.** Odds Ratios for Developing Hepatic Decompensation After Medical Addiction Therapy

Medical addiction therapy	Adjusted odds ratio (95% CI)	*P* value
Any pharmacotherapy	0.35 (0.23-0.53)	<.001
Naltrexone	0.27 (0.10-0.64)	.005
Gabapentin	0.36 (0.23-0.56)	<.001
Topiramate	0.43 (0.17-0.99)	.05
Baclofen	1.06 (0.39-2.69)	.91
Acamprosate	1.99 (0.99-4.059)	.06
Disulfiram	2.59 (0.54-13.26)	.24

When examining individual drugs, we found that the receipt of naltrexone (aOR, 0.27; 95% CI, 0.10-0.64; *P* = .005) or gabapentin (aOR, 0.36; 95% CI, 0.23-0.56; *P* < .001) were independently associated with reduced incidence of hepatic decompensation in patients with cirrhosis. Topiramate had no association with decreased likelihood of hepatic decompensation (aOR, 0.43; 95% CI, 0.17-0.99; *P* = .05). The receipt of baclofen, which was associated with reduced odds of developing ALD, was not associated with decreased likelihood of hepatic decompensation.

### Incidence of Hepatic Decompensation When Medical Addiction Therapy is Initiated After a Cirrhosis Diagnosis

We also sought to ascertain whether patients who initiated medical addiction therapy for AUD only after an index cirrhosis diagnosis was associated with decreased risk of future hepatic decompensation. We studied 105 patients with cirrhosis who were treated with medical addiction therapy after an index diagnosis of cirrhosis and 301 patients who did not receive medical addiction therapy; they were followed up for a mean (SD) duration of 11.8 (7.8) years and 8.6 (6.3) years, respectively, and then assessed for hepatic decompensation. We found that patients with cirrhosis who received medical addiction therapy after a diagnosis of cirrhosis were less likely to experience hepatic decompensation (aOR, 0.41; 95% CI, 0.23-0.71; *P* = .002). In a Cox proportional hazards regression, accounting for the time of follow-up, we found that patients in the treated group were less likely to experience a hepatic decompensating event than patients in the untreated group (HR, 0.38; 95% CI, 0.25-0.57; *P* < .001).

**Figure 2.**
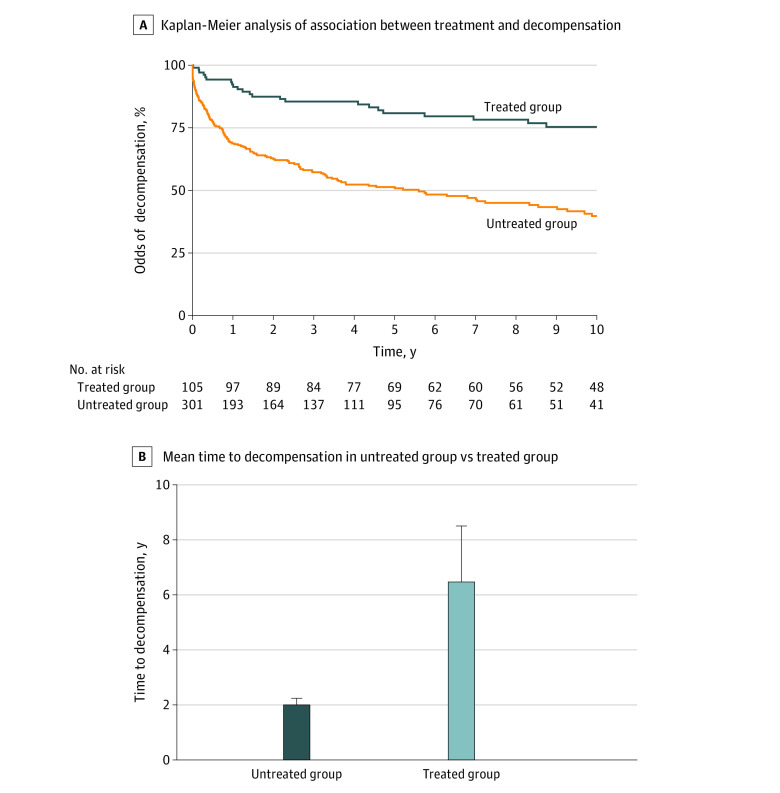
Association of Medical Addiction Therapy for Alcohol Use Disorder With Odds of Hepatic Decompensation Within 10 Years After Cirrhosis Diagnosis

In a Kaplan-Meier analysis, we found that the association of medical addiction therapy with reduced odds of hepatic decompensation persisted over the 10 years after index cirrhosis diagnosis (Figure 2). Furthermore, the mean (SD) time to hepatic decompensation after cirrhosis diagnosis was significantly longer in patients who experienced a hepatic decompensating event despite receiving medical addiction therapy compared with those who did not receive medical addiction therapy (6.3 (6.4) years vs 2.0 (3.5) years; *P* = .001) (Figure 2). Medical addiction therapy was initiated after a mean (SD) of 3.8 (5.1) years in the treated group. In a separate Kaplan-Meier analysis in which we counted the days free of hepatic decompensation starting from medical addiction therapy initiation as opposed to cirrhosis diagnosis, we found that the rate of hepatic decompensation at each time point in patients in the treated group was significantly lower than in patients in the untreated group (HR, 0.53; 95% CI, 0.39-0.72; *P* < .001).

## Discussion

In this retrospective study of a large, well-characterized cohort of patients with AUD, we found an association between receipt of medical addiction therapy and reduced odds of developing ALD. Furthermore, we found that patients with cirrhosis who received medical addiction therapy were less likely to develop a hepatic decompensating event over multiple years compared with those who did not receive such treatment. The associations of individual drugs with the outcomes of ALD and hepatic decompensation varied widely.

Little is known about the role of medical addiction therapy in preventing the development of ALD in patients with AUD. In this cohort study, patients with AUD who were treated with gabapentin, topiramate, or baclofen had the lowest odds of developing ALD during the follow-up period. These medications are used as off-label drugs for AUD but are not currently approved by the FDA for the treatment of AUD. Of all the therapies, gabapentin was found to be associated with the lowest odds of developing ALD. In addition to its alcohol-reducing properties, gabapentin is also known for its anxiolytic effects and its association with improved sleep quality,^25^ which are factors in the use of alcohol. Furthermore, we found novel evidence that supports the use of baclofen in preventing liver disease in patients with AUD. Given the well-recognized underuse of medical addiction therapy for patients with AUD,^26,27^ we believe the results of this study highlight the importance of medical addiction therapy in the setting of ALD.

We found that patients exposed to acamprosate had a higher likelihood of developing ALD. Acamprosate is widely prescribed to patients with AUD and liver disease given its favorable liver safety profile.^28^ However, compliance with acamprosate may be lower than with other medical addiction therapy medications because of its complex and frequent dosing regimen.^29,30,31^ In the present study, although noncompliance with acamprosate may diminish its potential benefit for the patient population, noncompliance alone would not explain its observed association with increased risk for ALD. It is possible that, given the favorable hepatotoxic profile of acamprosate, it is prescribed to patients who are deemed more likely to develop liver disease or have evidence of mild hepatic injury from alcohol. Furthermore, in our practice, acamprosate is reserved for use in patients with more severe AUD. Consistent with this explanation, more patients who received acamprosate had a diagnosis of severe AUD (defined as having both *ICD-10* diagnosis codes for conditions termed as *alcohol abuse* and *alcohol dependence*), compared with patients who received any other medication for AUD. Although the preponderance of evidence suggests that acamprosate may be beneficial for patients with AUD, a recent study found potential harm.^11^ A randomized clinical trial is needed to further elucidate this association between acamprosate and ALD development in well-matched patients with AUD.

In addition, we found that patients with alcohol-associated cirrhosis were less likely to experience a hepatic decompensating event if exposed to medical addiction therapy, even when such treatment was initiated only after their index cirrhosis diagnosis. This result is consistent with findings from previous studies of medical addiction therapy in patients with liver disease. Rogal and colleagues^20^ found that veterans with cirrhosis who were treated with FDA-approved medical addiction therapy had a 40% reduction in hepatic decompensation at 6 months. Similarly, Mellinger et al^21^ found reduced odds of hepatic decompensation at 1 year in patients with cirrhosis who received FDA-approved medical addiction therapy medications. Naltrexone is currently contraindicated in severe liver disease^32^ given the concern for hepatotoxicity and the precipitation of liver failure in these vulnerable patients. The results of this study suggest a benefit of naltrexone: it is a factor in not only preventing ALD but also limiting hepatic decompensation in patients with established cirrhosis. The data that support the risk of hepatotoxicity and liver failure in patients receiving naltrexone are limited. Accordingly, further studies examining the benefits and risks of naltrexone for the treatment of AUD in patients with ALD are needed.

### Limitations

This study has several limitations. First, this study was associative and possibly had unknown confounders. Second, the composition of the cohort was not balanced, with female individuals and racial and ethnic minority groups being underrepresented. Although we accounted for sex, race, and ethnicity in the multivariable analyses, the participants in the Biobank may not be representative of the general population. Third, the number of patients with AUD who received medical addiction therapy was higher than previously reported.^33^ This discrepancy may be explained by the availability of dedicated addiction services within the Mass General Brigham system and by the inclusion of off-label therapies for AUD. Fourth, adherence to AUD pharmacotherapy was difficult to assess. However, it is likely that this limitation would generate statistical noise and bias toward the null hypothesis and thus diminish the observed associations.

## Conclusions

The results of this cohort study show an association between receipt of medical addiction therapy and decreased likelihood of developing ALD in patients with AUD and lower incidence of hepatic decompensating events in patients with cirrhosis. The association between use of individual pharmacotherapy and incidence or progression of ALD and hepatic decompensation varied widely. In the absence of contraindications to medical addiction therapy, clinicians may consider the use of this treatment for AUD as a means to prevent ALD. Prospective randomized clinical trials are warranted to conclusively assess the benefits of both FDA-approved and off-label AUD pharmacotherapy as prophylaxis and treatment for ALD.

## Notes

Supplement. **eTable 1.** Definitions of All Variables Accounted for in Multivariable Analyses **eTable 2.** Further Demographic Data **eFigure.** Follow Up From Alcohol Use Disorder Diagnosis
