# Supplementary material for: Incidence and Progression of Alcohol-Associated Liver Disease After Medical Therapy for Alcohol Use Disorder
Source: JAMA Netw Open. 2022 May 20;5(5):e2213014. doi: 10.1001/jamanetworkopen.2022.13014 (PMC9123494; doi:10.1001/jamanetworkopen.2022.13014)
Supplement: Supplement. — eTable 1. Definitions of All Variables Accounted for in Multivariable Analyses eTable 2. Further Demographic Data eFigure. Follow Up From Alcohol Use Disorder Diagnosis [file jamanetwopen-e2213014-s001.pdf]

Supplementary Online Content

Vannier AGL, Shay JES, Fomin V, et al. Incidence and progression of alcohol-associated liver disease after medical therapy for alcohol use disorder. *JAMA Netw Open*. 2022;5(5):e2213014. doi:10.1001/jamanetworkopen.2022.13014

eTable 1. Definitions of All Variables Accounted for in Multivariable Analyses

eTable 2. Further Demographic Data

eFigure. Follow Up From Alcohol Use Disorder Diagnosis

This supplementary material has been provided by the authors to give readers additional information about their work.

© 2022 Vannier AGL et al. *JAMA Network Open.*

eTable 1. Definitions of All Variables Accounted for in Multivariable Analyses

Variable	Definition
**Age**	Continuous
**Sex**	M/F
**Race**	White/Black/Asian/Other/Unknown
**Hispanic Ethnicity**	Binary
**Weight Class**	Non-overweight, overweight, obese class I-III
**Nicotine Dependence**	ICD-10 code (F17.200)
**Hepatitis B**	ICD code (B18.0, B18.1) or a positive HBV antigen test or nonzero DNA test
**Hepatitis C**	ICD-10 code (B17.1, B18.2, B19.20) or a positive HCV antibody test, nonzero RNA test, or genotype test with a valid result)
**Homelessness**	ICD-10 code (Z59.0)
**Psychotherapy**	CPT4 code (90804-90815, 90826-90829, 90845, 90847, 90849, 90853, 90857, 90875, 90876, 99408)
**Cannabis Use Disorder**	ICD-10 code (F12.1, F12.2)
**Cocaine Use Disorder**	ICD-10 code (F14.1, F14.2)
**Inhalant Use Disorder**	ICD-10 code (F18.1, F18.2)
**Opioid Use Disorder**	ICD-10 code (F11.1, F11.2)
**Other Stimulant Use Disorder**	ICD-10 code (F15.1, F15.2)
**Sedative Use Disorder**	ICD-10 code (F13.1, F13.2)
**Anxiety Disorder**	ICD-10 code (F40-F48)
**Specific Personality Disorder**	ICD-10 code (F60)
**Mood Disorder**	ICD-10 code (F30-F39)
**Schizophrenia Disorder**	ICD-10 code (F20-F29)
**Chronic Passive Congestion of Liver**	ICD-10 code (K76.1)
**Hemochromatosis**	ICD-10 code (E83.11)
**Autoimmune Hepatitis**	ICD-10 code (K75.4)
**Primary Biliary Cirrhosis**	ICD-10 code (K74.3)
**Secondary Biliary Cirrhosis**	ICD-10 code (K74.4)
**Biliary Cirrhosis**	ICD-10 code (K74.5)
**Alpha-1 antitrypsin deficiency**	ICD-10 code (E88.01)
**Nonalcoholic Steatohepatitis**	ICD-10 code (K75.81)

© 2022 Vannier AGL et al. *JAMA Network Open.*

eTable 2. Further Demographic Data

Variable	All Patients (9635)	Any MAT (N=3906)	No MAT (N=5729)	P value†
**Hepatitis B, N (%)**	159 (1.6)	74 (1.8)	85 (1.4)	0.1224
**Hepatitis C, N (%)**	1149 (11.9)	613 (15.6)	536 (9.3)	<0.001
**Cannabis Use Disorder, N (%)**	1585 (16.4)	833 (21.3)	752 (13.1)	<0.001
**Cocaine Use Disorder, N (%)**	1467 (15.2)	890 (22.7)	577 (10.0)	<0.001
**Inhalant Use Disorder, N (%)**	1288 (13.3)	829 (21.2)	459 (8.0)	<0.001
**Opioid Use Disorder, N (%)**	1857 (19.2)	1186 (30.3)	671 (11.7)	<0.001
**Other Stimulant Use Disorder, N (%)**	512 (5.3)	344 (8.8)	168 (2.9)	<0.001
**Sedative Use Disorder, N (%)**	889 (9.2)	600 (15.3)	289 (5.0)	<0.001
**Anxiety Disorder, N (%)**	7250 (75.2)	3398 (86.9)	3852 (67.2)	<0.001
**Specific Personality Disorder, N (%)**	1140 (11.8)	726 (18.5)	414 (7.2)	<0.001
**Mood Disorder, N (%)**	7097 (73.6)	3384 (86.6)	3713 (64.8)	<0.001
**Schizophrenia Disorder, N (%)**	1432 (14.8)	779 (19.9)	653 (11.3)	<0.001

BMI: body mass index, MAT: medical addiction therapy, SUD: substance use disorder

†p values for categorical variables refer to the results of a Fisher exact test comparing patients receiving any MAT to those not receiving MAT.

© 2022 Vannier AGL et al. *JAMA Network Open.*

eFigure. Follow Up From Alcohol Use Disorder Diagnosis

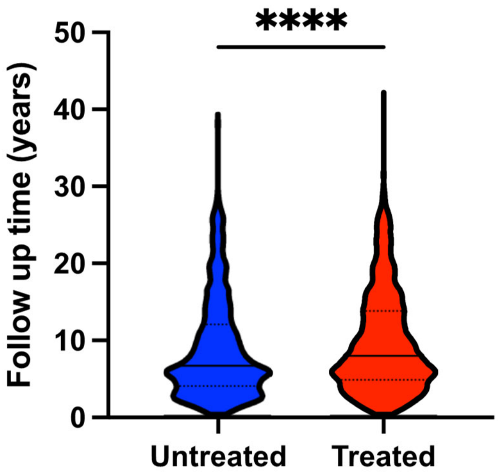

Violin plot representing the time of follow up since AUD diagnosis of patients that either received medical addiction therapy (MAT) (labelled as treated) or did not receive MAT (labelled as untreated). Patients receiving MAT had significantly longer follow up than patients that did not receive MAT (9.8 years vs 8.8 years, $p < 0.001$). Solid line represents the median, dotted line represents the 25th and 75th percentiles. ****: $p < 0.001$.

© 2022 Vannier AGL et al. *JAMA Network Open.*
